# Supplementary figures and images for: Serum cobalamin in children with moderate acute malnutrition in Burkina Faso: Secondary analysis of a randomized trial
Source: PLoS Med. 2022 Mar 9;19(3):e1003943. doi: 10.1371/journal.pmed.1003943 (PMC8906584; doi:10.1371/journal.pmed.1003943)

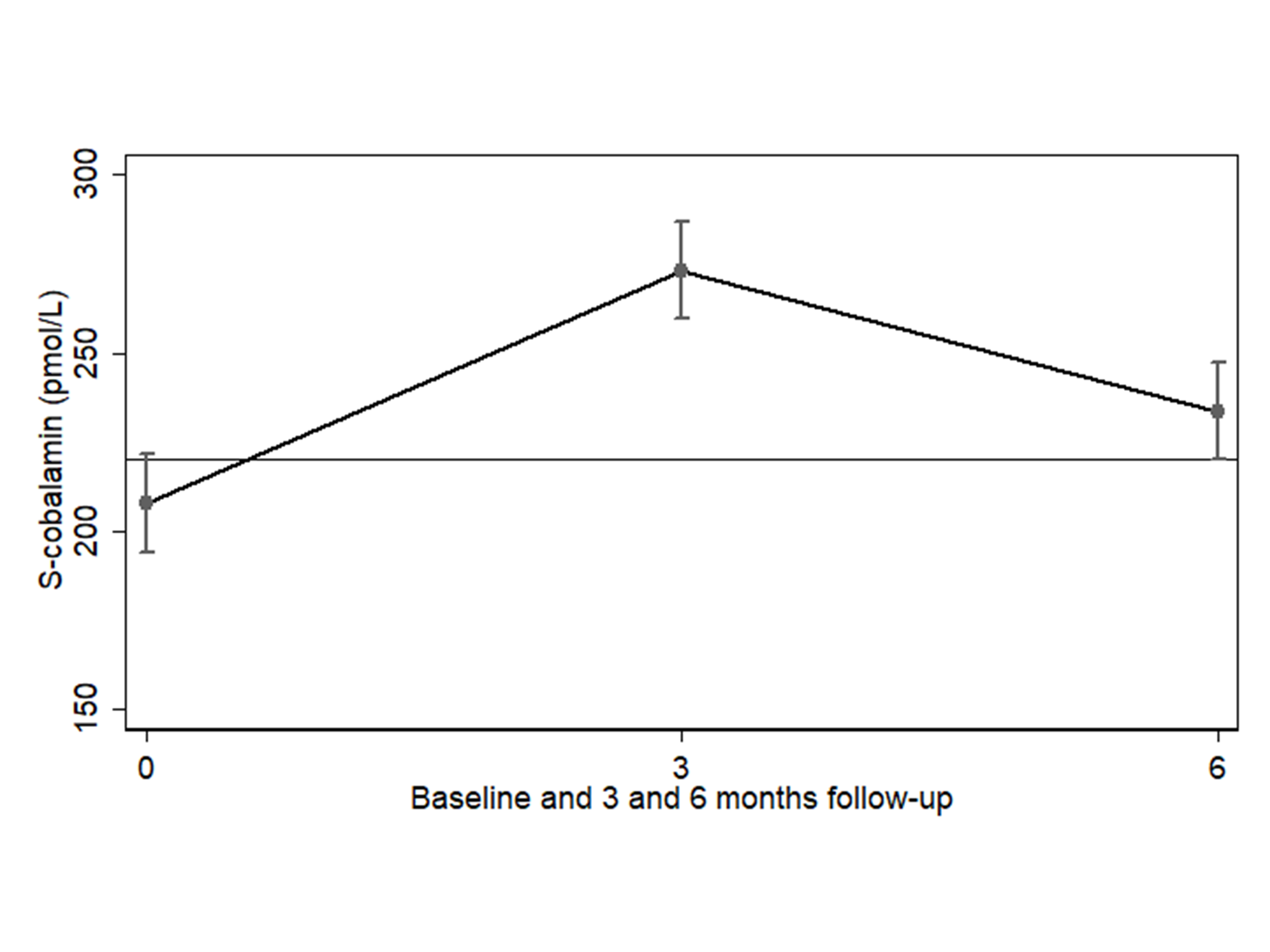

Supplement: S1 Fig — Mean (95% CI) SC at baseline, after 3 months supplementation, and after an additional 3 months without supplementation among 398 children with SC data at all time points. Based on tobit regression and adjusted for age, sex, months of admission, site and MUAC, WLZ and LAZ, inflammation and morbidity. Horizontal line at 222 pmol/L indicates cutoff to define normal SC. LAZ, length-for-age z-score; MUAC, mid-upper arm circumference; SC, serum cobalamin; WLZ, weight-for-length z-score. (TIF) [file pmed.1003943.s001.TIF]
