# Supplementary material for: Serum cobalamin in children with moderate acute malnutrition in Burkina Faso: Secondary analysis of a randomized trial
Source: PLoS Med. 2022 Mar 9;19(3):e1003943. doi: 10.1371/journal.pmed.1003943 (PMC8906584; doi:10.1371/journal.pmed.1003943)
Supplement: Attachment — Submitted filename: friis.pdf [file pmed.1003943.s004.pdf]

This paper reports on a trial of food supplementation in Burkina Faso.

I was asked for a statistical report and I interpret that to include all aspects of the design and conduct of the study.

Points of detail

Page 2 I think SC is missing from the helpful list of abbreviations.

Page 4 I think it is better to say that the authors were unable to find any effect of milk. Absence of evidence is not evidence of absence.

Page 6 The inclusion and exclusion criteria are not well specified here. What cut–off for MUAC was used? How were MUAC and WLZ combined to form a cut–off?

Page 6 What exactly is the meaning behind the sentence starting 'The final assessment'? Does this mean they applied yet another set of criteria?

Page 7 Looking at the title of reference 10 it seems this is a validation in Burkina Faso. That is an important fact so I would mention that specifically here to save readers having to seek the information elsewhere. A sentence or two summarising the results of reference 10 would not go amiss either.

Page 8 Using existing cut–off points for SC is understandable but it does raise the issue of whether the interesting effects occur at exactly those points. Looking at the shape of the relationship between SC as a continuous variable might be enlightening.

Page 8 I suspect only Stata users will understand what xtmixed does. A description of the model in English as given later would be more widely understood.

Page 10 A major omission here is the so–called CONSORT diagram giving us the low of participants through the study (Begg et al., 1996; Moher et al., 2010). This is important here since Table 1 shows 1192, 1330 and 399 children with data at each of the three time points. This flow of children through the study is surprising but we are not told why or how this has happened. We also do not know why about a quarter of children lacked baseline values although we do see the correlates of missingness. All those correlates should be included in the modelling.

Page 10 The authors have imputed a value for those below the limit of detection but this makes the means uninterpretable. The position is worse

for the standard deviations as imputing a constant under–estimates variability. Medians and inter–quartile ranges would be better.

Page 10 and following Here and occasionally later we just see a p–value instead of the usual effect size and its confidence interval. Effects which fail to reach some arbitrary level of statistical significance still need fully reporting.

Page 11 Data not shown could go into the supplementary material if the authors feel it would break up the narrative too much.

Points of more substance

Missingness

I do not think the figure is very helpful in the light of the differences in the numbers available at each time point. If we are interested in trajectories over time then more clarity is needed about exactly what analysis was done in xtmixed and a different graphic would be beneficial. A spaghetti plot would seem useful or perhaps several stratified by the important treatment variables. If the plot is too cluttered just plot a random sub–sample.

Limitations

The authors do not mention the issue of whether the index child consumed the supplement. Unless mothers in Burkina Faso are very different from those in the UK I would have thought that in conditions of food insecurity she would share food between her children. Do the authors have any data on actual consumption? To be fair this point applies to all real–world studies of supplementation.

Data ownership

I am not a lawyer but how does it happen that data on people in Burkina Faso, collected in Burkina Faso, becomes the exclusive property of Denmark? I could understand if it was subject to the relevant privacy law in Burkina Faso. I suppose I may be more sensitive to such issues coming from a country with a long history of removing the population and resources of Africa for our benefit

Summary

Some concerns about the analysis. The article seems rather under–reported but perhaps when the authors come to provide the CONSORT checklist that will help reveal missing detail.

Michael Dewey

References

C Begg, M Cho, S Eastwood, R Horton, D Moher, I Olkin, R Pitkin, D Rennie, K F Schulz, D Simel, and D F Stroup. Improving the quality of reporting of randomized controlled trials: the CONSORT statement. *Journal of the American Medical Association*, 276(8):637–639, 1996.

D Moher, S Hopewell, K F Schulz, V Montori, P C Gøtzsche, P J Devereaux, D Elbourne, M Egger, and D G Altman. CONSORT 2010 explanation and elaboration: updated guidelines for reporting parallel group randomised trials. *British Medical Journal*, 340(c869), 2010.
