# Supplementary material for: Serum cobalamin in children with moderate acute malnutrition in Burkina Faso: Secondary analysis of a randomized trial
Source: PLoS Med. 2022 Mar 9;19(3):e1003943. doi: 10.1371/journal.pmed.1003943 (PMC8906584; doi:10.1371/journal.pmed.1003943)
Supplement: Attachment — Submitted filename: Friis-Cobalamin-Response-letter-22Dec21-final.docx [file pmed.1003943.s005.docx]

**Point-to-point response to editor and review comments**

**Requests from the editors:**

1)	Please revise your title according to PLOS Medicine's style. Your title must be nondeclarative and not a question. It should begin with main concept if possible. For example, please place the study design in the subtitle (i.e., after a colon). For example, “Serum cobalamin in children with moderate acute malnutrition in Burkina Faso: A cross-sectional study of findings from a randomized trial” .

**Response:** Thanks. We have moved Burkina Faso up. As for the design, our study contains findings from both cross-sectional, longitudinal and RCT analyses, so we think only mentioning “a cross-sectional study” will be misleading. We therefore suggest “*secondary analysis of a randomized trial*” as the most correct. Then we mention the overall study design in the title, ie the RCT, and by adding “secondary analysis” we also cover the other designs. We hope this is acceptable, otherwise please advise.

2)	Please delete the “RESEARCH IN CONTEXT” section – this is not required by PLOS Medicine

**Response:** This has been deleted

3)	At this stage, we ask that you write a non-technical Author Summary. The Author Summary should immediately follow the Abstract in your revised manuscript. This text is subject to editorial change and should be distinct from the scientific abstract. The summary should be accessible to a wide audience that includes both scientists and non-scientists. Please see our author guidelines for more information:

**Response:** Author Summary has been included

4)	Abstract:

a)	Please report your abstract according to CONSORT for abstracts: <https://eur02.safelinks.protection.outlook.com/?url=http%3A%2F%2Fwww.consort-statement.org%2Fextensions%3FContentWidgetId%3D562&amp;data=04%7C01%7Chfr%40nexs.ku.dk%7Cfc63b07447e44cf44f0208d98a7aae21%7Ca3927f91cda14696af898c9f1ceffa91%7C0%7C0%7C637693081931505846%7CUnknown%7CTWFpbGZsb3d8eyJWIjoiMC4wLjAwMDAiLCJQIjoiV2luMzIiLCJBTiI6Ik1haWwiLCJXVCI6Mn0%3D%7C1000&amp;sdata=R2ZpJcGtbWKLWnvwIkQ0%2BVZFrwMcmcGlK5PxEMKlWkM%3D&amp;reserved=0> .

**Response**: please note that based on this link, the CONSORT for Abstract is relevant “*… when reporting the main results of a randomized trial …*”. As mentioned in our cover letter, and as acknowledged by reviewers – and by the editors in the comment about the title - this manus is not reporting the main results of a trial. In contrast, we report the prevalence and correlates of serum cobalamin based on cross-sectional data, then we report the overall change in cobalamin based on longitudinal data, and finally the effects of the interventions from a RCT, but only on serum cobalamin, which was not the primary outcome. However, we has revised the manus also contains the information required, but also the findings from the other analyses. As such, we have included information about adverse events, although this was already published.

Structure your abstract using the PLOS Medicine headings (Background, Methods and Findings, Conclusions).

**Response**: this has been corrected

b)	Please combine the Methods and Findings sections into one section, “Methods and findings”. Replace “Interpretation” with “Conclusions”

**Response**: this has been corrected.

c)	Please ensure that all numbers presented in the abstract are present and identical to numbers presented in the main manuscript text.

**Response**: this has been checked.

d)	Please quantify all results (with p values in addition to 95% CI).

**Response**: this has been corrected

e)	Please include a summary of adverse events if these were assessed in the study.

**Response**: We included this when reporting the main effects of the interventions in the RCT in Fabiansen et al, 2017. However, we have now added the following to the result section in the abstract (L76-7):

“*Four children died and no child developed an allergic reaction to supplements*”

In addition, we have added the following to the main text in the method section (L151):

“*Serious adverse events were defined as anaphylaxis and death*”

and the result section (L274-5):

“*As reported previously, four children died and no child developed an allergic reaction to supplements (ref)*”

f)	In the last sentence of the Abstract Methods and Findings section, please describe the main limitation(s) of the study's methodology.

**Response**: we have added the following (L77-8):

“*The main limitations of this study was that only serum cobalamin was available as a marker of status and was missing from a quarter of the children*”.

5)	Did your study have a prospective protocol or analysis plan? Please state this (either way) early in the Methods section:

a) If a prospective analysis plan (from your funding proposal, IRB or other ethics committee submission, study protocol, or other planning document written before analyzing the data) was used in designing the study, please include the relevant prospectively written document with your revised manuscript as a Supporting Information file to be published alongside your study, and cite it in the Methods section. A legend for this file should be included at the end of your manuscript.

b) If no such document exists, please make sure that the Methods section transparently describes when analyses were planned, and when/why any data-driven changes to analyses took place.

c) In either case, changes in the analysis-- including those made in response to peer review comments-- should be identified as such in the Methods section of the paper, with rationale.

**Response**: serum cobalamin was not explicitly mentioned as an outcome in the trial registration, hence was not included in our analysis plan. It was only when reporting our data on child development that we realized the need for data on cobalamin and subsequently found resources to have it analysed from stored samples. We have made this clear by including the following in the method section (L230-33):

“*Serum cobalamin was not a pre-planned outcome of the trial, however, we specified which covariates to assess as potential correlates and confounders before we received the cobalamin data and performed the statistical analyses*”.

6)	Please ensure that the study is reported according to the STROBE guideline for observational studies, and include the completed STROBE checklist as Supporting Information. Please add the following statement, or similar, to the Methods: "This study is reported as per the Strengthening the Reporting of Observational Studies in Epidemiology (STROBE) guideline (S1 Checklist)." The STROBE guideline can be found here:

**Response**: while this paper presents data based on both observational studies and RCT, we have tried to report relevant items of both STROBE and CONSORT, and uploaded check lists for both.

7)	Your study is observational and therefore causality cannot be inferred. Please remove language that implies causality throughout the manuscript, such as “effect/s.” Refer to associations instead.

**Response**: this has been done, except where we give the estimates of the effects of the interventions based on the RCT.

8)	In the Methods and Results section, please consistently provide 95% CIs and p values for estimates in the main text and tables.

**Response**: this has been corrected wherever we report a difference or association. However, where we give mean serum cobalamin with 95%CI for descriptive purposes only, then obviously a p-value is meaningless and has not been included.

9)	In table 5 and 6, please provide the meaning of the bolded numbers in the footnotes.

**Response**: since we added p-values in response to the previous comment we have now removed the bolding.

10)	Please provide the meaning of bars and whiskers in your figures.

**Response**: in Figure 2 (and our new Supplemental figure) we do indicate that what are shown are means (95%CI), so we believe it should be clear.

11)	Please replace the terms “girls” with “females,” and “boys” with “males”

**Response**: this has been corrected

12)	Please replace the term “marginally significant” with “not significant”

**Response**: this has been corrected

13)	Please remove the ‘Funding’, from the end of the main text. In the event of publication, this information will be published as metadata based on your responses to the submission form.

**Response**: this has been corrected

14)	Please use the "PLOS Medicine" citation style for reference formatting and see our website for other reference guidelines a)	Please ensure that only six names precede et al.

b)	Remove the “Public Library of Science” from ref #7

**Response**: we have changed citation style, and corrected ref #7.

Comments from the reviewers:

**Reviewer #1:**

This is an important report of secondary data analysis of an RCT with results published in 2017 in this journal. There are other analyses from this same data (well cited in this manuscript) however the authors do not tell us relevant findings from those in the background. Here the authors look at the i) level and the determinants/correlates of cobalamin serum levels (and haemoglobin and child development) at the baseline of trial, and ii) the effects of food supplementation (matrix, soy quality and milk content) on serum cobalamin during the follow-up. The analysis is not conducted as in an RCT. Interaction coefficients were decided upon a p-value calculation. It is a well-written report however there are a few issues to be addressed before publication.

Major issue:

- I have to commend the authors to conduct a Tobit regression for the multivariable analysis of the serum cobalamin to address the fact that measurement is left censored. However, some of the analysis was conducted with the replacement of these censored values with 112/√2. This is an arbitrary choice. Did you conduct sensitivity analysis with another value? Why not use Tobit when comparing this serum cobalamin in all analyses rather than the t-tests or ANOVA?

**Response**: Thanks a lot for this very helpful comment. We now avoid the need for replacement of censored values by presenting medians (IQR) instead of means (SD) for descriptive purposes, and by using tobit in all the analysis with serum cobalamin as outcome.

As such, we use tobit regression for the analysis behind Table 2 and 3 (correlates of baseline serum cobalamin), and for the analysis behind table 6 (effects of food ingredients) in which we adjust for baseline serum cobalamin as a separate variable. We use a tobit model (with normally distributed random effects, ie xttobit in Stata) in the analysis with repeated measures, ie where we look at the overall change over the three time points, with the group/panel variable specified using xtset. Based on other reviewer comments we now also adjust these analysis for “correlates of missing”).

So, we have made major changes to the description of the analyses in the method section (L235-52). Accordingly, the data in text, tables and figure have been updated, although there only slight differences. In addition, we now also mention that we use tobit regression under table 6 and Figure 2.

Minor issues:

- Line 145 better to write weeks rather than wk

**Response**: this has been corrected (2 occurences)

- Line 151 please cite the zscore06 package and tell us the version of these WHO reference tables

**Response**: we now include a proper reference for zscore06. As for the tables, we have now specified that we used the simplified field tables and added a reference to the WHO homepage. (L184-5)

- Line 151 cite properly Stata 12; and line 189 Stata 14.

**Response**: this has been corrected to Stata 14, and we now cite in the text (L185-6 and L229-30) as (StataCorp. 2015. Stata Statistical Software: Release 14. College Station, TX: StataCorp LP).

- Line 188 cite properly the Epidata 3.1 software

**Response**: we now cite in the text (L228) as: (Epidata Association, Odense, Denmark)

- Line 158 please add a justification for using Malawian MDAT z-scores for Burkina Faso children

**Response**: Since the MDAT is developed in Malawi, but intended for children in sub-Saharan Africa, it is more culturally appropriate than commonly used instruments such as Bayley scales which are designed for children from high-income countries. Furthermore, we have adapted the MDAT to BF, and validated and piloted it there. In brief, we found that the tool was appropriate in the setting, it had a high inter- and intra-rater reliability and sensitivity and it was well accepted by study participants.

We have added a couple of words to make that more clear (L188-9):

“*Adaptation, validation, and pilot testing of the MDAT tool have been reported previously (ref).*”

- Line 199 please write "linear mixed-effects models" rather than "xtmixed models". Xtmixed is a Stata command therefore only a few Stata users would recognise it.

**Response**: Thanks. We have revised the analyses and now use tobit and mixed-effects tobit regression instead.

- Line 233 I think it would be better if it is written "level and correlates of baseline serum cobalamin".

**Response**: this has been corrected (L294).

- There is an important amount of missing data on cobalamin. For the longitudinal data analysis, the linear mixed-effects analysis implicitly deals with missing data under Missing at Random assumption (I think that is fine). At the baseline, the randomization may have offered the MCAR (missing completely at - random) so it is correct to ignore the missed measurements (although table 1 offers some concerning p-values at baseline). I think it would be good to discuss the data missing in strengths/limitations.

**Response**: Thanks a lot for this very helpful comment. We’ve revised the discussion accordingly (L463-71):

*“The main limitation is missing data on serum cobalamin, mainly due to lack of serum. For the longitudinal data, missing data were associated with age, morbidity, inflammation and haemoglobin, which may indicate that data were missing at random (i.e. missing data might be predicted by observed data). Consequently, analyses using mixed-effects models will still provide unbiased results. The observation is further corroborated by results from analyses that included adjustment for correlates of missingness and, also, complete-case analyses only including children with data at all three time points; all these analyses provided very similar findings. For the cross-sectional analyses, missing values were also associated with age and inflammation and, therefore, only results from analyses adjusted for correlates of missing were reported”*.

**Reviewer #2:**

Thank you very much for asking me to look at this work. In general, this is a very well written manuscript and follows on several papers that have been written from the clinical trial

There are a number of minor issues worthy of comment here:

1.	The paper combines two study designs: a cross sectional study and a randomized controlled trial. Clearly a cross sectional study has its own limitations since the exposure and outcome are being are studied at the same time.

**Response**: We agree. This is described as a limitation in L473.

2.	The second issue is that of the sample size for the study. This section is missing in this manuscript. The basis for the sample size including the effect size, power and precision is missing.

**Response**: we have added the following text to describe the sample size considerations (L222-6):

“*As previously reported, the sample size was calculated for the primary outcome of the Treatfood trial [5]. With a sample size of 1608 (i.e. 134 in each of the 12 combinations resulting from the 2x2x3 factorial design) we had 80% power to detect a 0.6 SD difference in any pairwise comparison with 5% significance level, while allowing for 20% loss to follow-up. If no interaction, the sample size would allow detection of smaller effects”*.

3.	While the objectives were clearly stated, the RCT should have had a clear hypothesis regarding the intervention and cobalamin etc.

**Response**: we already write the objective of the main Treatfood trial (L125-7), as well as the three aims of the current paper (L130-33). To be frank, we didn’t have explicit hypotheses about effects of the experimental interventions on cobalamin status. While milk was the only ingredient containing cobalamin, we knew that the contribution from milk was modest compared to the premix added to all products. As for the effects of LNS vs CSB and soy isolate vs dehulled soy, we knew that there was no difference in cobalamin content, and had no pre-specified hypothesis about effects on bioavailability. So, while we thought it would be wrong to specify hypothesis, we thought we should assess the effects given the strong design.

4.	The process of randomization was not adequately described in the manuscript. This includes the process itself, allocation concealment etc. It is possible that the process was described in more detail elsewhere in the papers preceding this one, so the authors could describe it to a certain extent and refer to the detailed description in the previous papers from the same work.

**Response**: we agree, this information should be given. We have included the following text (L168-75):

“*As described previously (Ref), we individually randomized children to 1 of 12 combinations of the three factors, with variable block size and stratified by site, using www.randomization.com by a person not otherwise involved in the trial. The supplements were designated by a 1-letter code by the manufacturer, which was placed in a 12-letter sequence labelled on each supplement. The code key was kept in a sealed envelope in a safe until study completion. Only one person, otherwise not involved in the study, was aware of the random sequence and code system and relabeled supplements with individual study identifications. Study participants, outcome assessors, and other study staff were blinded with respect to soy quality, milk content and matrix, while it was not possible to blind study participants with respect to matrix*”.

5.	Why were cobalamin results available for only 1192/1609 children? Despite this comment, the authors have clearly compared those with the data and those without. This is commendable.

**Response**: Thanks. We agree this should have been better explained. Please see our response to similar comment from reviewer #1 above.

6.	The issue of other infections other than bacteria were not considered in detail. Is it possible that other infections such as Ascaris lumbricoides, hookworm and HIV affected the baseline levels? We note that the children were give albendazole at the beginning of the study. Be that as it may, it would appear that this is an area this is an area needing further study.

**Response**: unfortunately, we don’t have data on other infections. We know that the prevalence of HIV among women of reproductive age was <1%, so it is likely to be negligible in small children. We agree that the contribution of helminths and other parasitic infections to cobalamin status – and to environmental enteric dysfunction – should be studied further.

7.	There is need for study profile

**Response**: Thanks a lot. A study profile has now been included as figure 1.

8.	The authors have published part of this work [1] but without looking at cobalamin. In that paper the authors assessed gross motor, fine motor, language and cognitive skills. It is not clear why the current paper has not looked at cognitive function and cobalamin deficiency.

References

1.	Olsen MF et al (2020) Impact of food supplements on early child development in children with moderate acute malnutrition: A randomised 2 x 2 x 3 factorial trial in Burkina Faso. PLOS Medicine 17(12): e1003442.

**Response**: Yes, data on cobalamin were not available when publishing the paper by Olsen et al mentioned above. But, we realized that cobalamin would be important to study, and then found funds to make it possible. We have added the following sentence to make this clear (L127-8):

“We have previously reported that food supplements were beneficial for child development which motivated us to assess the role of cobalamin”.

**Reviewer #3:**

General

The objective of this study was to describe the prevalence of low vitamin B12 status (serum cobalamin, SC) among Burkinabé children 6-23 mo of age with moderate acute malnutrition (MAM) participating in a food supplementation trial, and examine the impact of various food supplements on SC. As noted by the authors, there is a general lack of information on B12 status among children in Africa, and particularly among children with MAM. This paper thus adds new information on vitamin B12 deficiency and the effects of food supplements on micronutrient status in this population. In general the paper is well written and methods are appropriate.

There was a relatively high proportion of missing baseline SC values (26%). The authors' analysis of characteristics of children with available vs missing samples provides insight on the possible direction of bias. However, I do not see a comparison of whether/how missing samples differed by intervention group. This would be helpful to report as differential missing values by group could potentially influence the conclusions on the intervention effects.

**Response**: Thanks for drawing our attention to this. We have now included a trial profile (as Figure 1), where we show the number of children with cobalamin data at each visit, and by experimental intervention. In addition, we have included the following sentence in the result section (L291-92):

“*Missing serum cobalamin was not associated with any of the three interventions at baseline, 3 months or 6 months (all p>0.18, Figure 1)*”.

The abstract concludes that "The SC increase during supplementation was inadequate. The bioavailability and adequacy of cobalamin in food supplements should be reconsidered." And in Lines 361-364: "The overall increase in serum cobalamin during supplementation was insufficient as more than one-third of the children were left with marginal or low serum cobalamin. Furthermore, during the subsequent 3 months there was a considerable decline. This suggests that the bioavailability of cobalamin is compromised in children with MAM."

The observation that a large proportion still had low SC after 3 months of treatment is important and understanding the role of bioavailability would be useful. I wonder if another explanation (just speculation) might be that B12 is diverted quickly to functional sites (including bone marrow) during repletion so that less remains in circulation compared to a person with adequate B12 status.

However, I disagree that the interpretation of the decrease after 3 months of no supplementation signals a bioavailability problem. This decrease is not surprising as SC concentrations are responsive to recent intake and would be expected to decline if children discontinue supplemental food are returning to a diet that is very low in animal-sourced foods or other sources of B12.

**Response**: Agree, the sentence “This suggest that the bioavailability of cobalamin is compromised” has now been deleted. As the reviewer writes, the decline merely reflects a reduction in daily intake, with continued requirement and diversion of cobalamin to target tissues.

So, we have also added the following (L445-6):

“*… reflecting low intake and continued diversion of cobalamin to target tissues*”.

Minor

Lines 126-128: Any relevant exclusion criteria?

**Response**: We have now given a full description of inclusion and exclusion criteria by replacing the second to last sentences under “Study design, area and participants” with the following text (L142-8):

“*Children between 6-24 months who were residents in the catchment area were recruited if a diagnosis of MAM was confirmed, i.e., MUAC 115 to <125 mm and/or WHZ -3 to <-2 in the absence of oedema. Children were excluded if treated for severe acute malnutritioin (ie WHZ <−3 or MUAC< 115 mm or oedema) or hospitalised within the past 2 months, if already in a nutritional programme, or (requiring hospitalisation, e.g., haemoglobin <50 g/l. Children with a severe disability, limiting the feasibility of investigations, or with suspected allergy to milk, peanuts, CSB, or LNS were also excluded*”.

Lines 140-141: "The cobalamin content from premix was 4.1 μg in CSB and 3.2 μg in LNS." Is this the content per recommended serving?

**Response**: thanks, we agree this was not clear. We have now clarified that this was “per daily ration” (L163).

The detection limit of 112 pmol/L seems somewhat high compared with the cutoff for deficiency <149 pmol/L.

**Response**: we believe this is normal, and reflect that if serum cobalamin is low (or if clinical suspicion) then serum MMA will be measured as the specific marker of deficiency/diagnostic test.

Methods: What year(s) was the study carried out vs what year was SC measured? That is, how long were samples stored, and under what conditions, prior to analysis? This would be especially useful since the outcome is not specifically listed in the trial registration.

**Response**: thanks, we agree this was not clear. We have now added that the Treatfood trial was “*conducted from September 2013 to August 2014*” (L272). We have also added that the sample were stored “*at -80°C until analysis*” (L205). The samples were kept at -80°C for 6-7 years. There are data showing serum cobalamin levels remain largely unchanged at -25°C for up to 29 years (Hannisdal et al. Journal of Nutrition, 2020).

Lines 194-196 and 199-200 and 206-207: How was multicollinearity assessed in the models (especially if multiple anthropometric measures were used in the same model?)

**Response**: L194-196 refers to analysis only adjusted for age and sex. As for the other analyses, we were not interested in assessing the effects of these anthropometric variables, in which case we agree collinearity would have been a problem. We only used multiple anthropometric measures to ensure that confounding was adequately adjusted for. We acknowledge that these variables will be correlated, but believe the advantage achieved in terms of reducing confounding outweighs the slight change in standard errors due to multicollinearity. In fact, we have now run the model with one and all anthropometric variables, and it doesn’t change the SE.

Minor note: Line 200 refers to WHZ while line 206 refers to WLZ.

**Response**: since children were below 24 months at admission and were measured lying we have corrected it to WLZ throughout (2 occurrences).

Lines 303-305: For associations between B12 and inflammation, please see this recent paper by the BRINDA group (Young et al, AJCN 2020). This study also found no association in multiple cross-sectional surveys.

**Response**: thanks a lot for this. We have now added this reference (Ref#23/L382).

Lines 353-355: What information is available on the stability of B12 in LNS or CSB? Are there data to suggest that stability is lower in CSB?

**Response**: while this is a good point, we have not been able to find any reference on this.

**Reviewer #4:** (comments copy-pasted from pdf).

Page 2 I think SC is missing from the helpful list of abbreviations.

**Response**: SC has now been added to the list.

Page 4 I think it is better to say that the authors were unable to find any effect of milk. Absence of evidence is not evidence of absence.

**Response**: Correct, we have now changed the sentence in the abstract to (L76) “No effect of milk was found”.

Page 6 The inclusion and exclusion criteria are not well specified here. What cutoff for MUAC was used? How were MUAC and WLZ combined to form a cutoff?

**Response**: Thanks, we agree this was not clear. We have now given a full description of inclusion and exclusion criteria by replacing the second to last sentences under “Study design, area and participants” (L142-8), as mentioned above.

Page 6 What exactly is the meaning behind the sentence starting `The final assessment'? Does this mean they applied yet another set of criteria?

**Response**: The initial assessment was a screening by community health workers and screening teams. The final assessment was made by the trained research staff at study sites, using properly computed WHZ (rather than based on simplified field tables), as well as the whole set of inclusion and exclusion criteria, which we now describe in detail.

Page 7 Looking at the title of reference 10 it seems this is a validation in Burkina Faso. That is an important fact so I would mention that specifically here to save readers having to seek the information elsewhere. A sentence or two summarising the results of reference 10 would not go amiss either.

**Response**: This is an important point, also raised by reviewer #1. Please see our response above. We found that the tool was appropriate in the setting, it had a high inter- and intra-rater reliability and sensitivity and it was well accepted by study participants. We are not sure this sentence belong to a method section, but hope it is implicit in the sentence we slightly reworded (L188-9).

Page 8 Using existing cutoff points for SC is understandable but it does raise the issue of whether the interesting effects occur at exactly those points. Looking at the shape of the relationship between SC as a continuous variable might be enlightening.

**Response**: Yes, it is indeed a major issue in cobalamin research that the sensitivity and specificity of cobalamin and serum methylmalonic acid (MMA), the only specific marker of cobalamin deficiency, are not well-defined. Most research on this has been done in relation to pernicious anaemia. While this could also be interesting with respect to other cobalamin-dependent functions, we think this should be done in a different study, where data on MMA are available.

Page 8 I suspect only Stata users will understand what xtmixed does. A description of the model in English as given later would be more widely understood.

**Response**: thanks, but we have revised the analyses and now use tobit regression, based on other reviewer comments.

Page 10 A major omission here is the so{called CONSORT diagram giving us the low of participants through the study (Begg et al., 1996; Moher et al., 2010). This is important here since Table 1 shows 1192, 1330 and 399 children with data at each of the three time points. This flow of children through the study is surprising but we are not told why or how this has happened. We also do not know why about a quarter of children lacked baseline values although we do see the correlates of missingness. All those correlates should be included in the modelling.

**Response**: Thanks a lot. We have now added a flow chart (Figure 1), and also adjusted for correlates of missingness in the model describing changes over time in mean SC, and included the following (L328-9):

“*…as well as inflammation, morbidity, and haemoglobin, found to be correlates of missing cobalamin data at 6 months (Table 1)*”.

The change over time shown in the Figure (now Figure 2) has been updated, and we added a Supplemental figure showing the same changes among the 310 children with data at all three time points.

Page 10 The authors have imputed a value for those below the limit of detection but this makes the means uninterpretable. The position is worse for the standard deviations as imputing a constant underestimates variability. Medians and inter-quartile ranges would be better.

**Response**: Thanks a lot. As mentioned in response to comments above we now give medians (IQR) rather than means for descriptive purposes. In addition, we don’t impute values for those below limit of detection, but instead use tobit regression.

Page 10 and following Here and occasionally later we just see a p-value instead of the usual effect size and its confidence interval. Effects which fail to reach some arbitrary level of statistical significance still need fully reporting.

**Response**: The reviewer refer to text like this: “The 417 children without baseline serum cobalamin were younger (p<0.001), had higher serum concentrations of inflammatory markers (p<0.03), and were more likely to be breastfed (p=0.04), but there were no differences with respect to sex distribution, anthropometry, morbidity, hemoglobin and measures of child development (**Table 1**).” Since we just want to highlight and summarize findings from the table about missing data, we think it will be too much to repeat effect sizes in the text. We have therefore deleted the p-values and just refer to the table.

Page 11 Data not shown could go into the supplementary material if the authors feel it would break up the narrative too much.

**Response**: Thanks a lot. We have now made a supplementary figure instead. See also our response to missingness, below.

Points of more substance

Missingness:

I do not think the figure is very helpful in the light of the differences in the numbers available at each time point. If we are interested in trajectories over time then more clarity is needed about exactly what analysis was done in xtmixed and a different graphic would be beneficial. A spaghetti plot would seem useful or perhaps several stratified by the important treatment variables. If the plot is too cluttered just plot a random sub-sample.

**Response**: We agree that the difference in numbers is a problem. However, we have followed the advice from the reviewer to adjust the analysis for correlates of missingness (see also response above), and then we have added a (supplemental) figure based on 310 children with data at all three time points. With this we hope the reviewer can accept that we keep the figure, since we believe it gives important information.

Limitations:

The authors do not mention the issue of whether the index child consumed the supplement. Unless mothers in Burkina Faso are very different from those in the UK I would have thought that in conditions of food insecurity she would share food between her children. Do the authors have any data on actual consumption? To be fair this point applies to all real-world studies of supplementation.

**Response**: we agree this is an important limitation in all food supplementation trials. We have added a the following text in the method section (L148-50): “*In order to reduce mixing or sharing of the supplements, we only included the first child with MAM identified from a household, but provided the same supplement to siblings with MAM, as well as to discordant twins*”.

Data ownership:

I am not a lawyer but how does it happen that data on people in Burkina Faso, collected in Burkina Faso, becomes the exclusive property of Denmark? I could understand if it was subject to the relevant privacy law in Burkina Faso. I suppose I may be more sensitive to such issues coming from a country with a long history of removing the population and resources of Africa for our benefit.

**Response**: Thanks, the point is well taken. The reviewer refer to the statement at page two, “*Data described in the manuscript, code book, and analytic code will not be made available because the Danish Act on Data Protection does not allow for personal data to be made available to others without prior individual approval from the Danish Data Protection Agency*”. This is the (short version of the) text we are told to use by our University due to GDPR, etc. In addition, we didn’t obtain permission by the ethics committee and participants in BF to share data.

We would have liked to share, and we agree it is not satisfactory - and looks odd.

We usually do our research through long-term collaboration with research institutions in various countries in Africa, with an emphasis on capacity building, including PhD-training. This trial is an exception, because it started as a collaboration between Doctors without Borders (DK) and University of Copenhagen, with an intention to conduct the trial in a humanitarian setting. Eventually, we established a collaboration with ALIMA, and the trial was conducted within their programme. This is the reason why we don’t have a local PI from a research institution in Burkina Faso. We planned PhD-training for two young researchers, but only succeeded to complete one. We regret that only one researcher from Burkina Faso was involved as coauthor.

In addition to the revision arising from the reviewer comments, we also added a sentence about external validity to comply with the STROBE/CONSORT check lists (L460-61):

“The findings are likely to be generalizable to children with MAM in low-income settings in Sub-Saharan Africa, with limited access to animal-source and fortified foods”.

L60-61:

“randomisation resulted in baseline equivalence between intervention groups”

L459-60:

”As previously reported, randomisation resulted in baseline equivalence between intervention groups (ref).”
